# A28 A SERUM REDOX STATUS-RELATED INDICATOR IS ASSOCIATED WITH THE RISK OF ONSET OF CROHN'S DISEASE

**DOI:** 10.1093/jcag/gwad061.028

**Published:** 2024-02-14

**Authors:** K Mu, M Xue, W Turpin, K Croitoru

**Affiliations:** . medicine, University of Toronto, Toronto, ON, Canada; . lunenfeld-tanenbaum research institute, Mount Sinai Health System, Toronto, Toronto, ON, Canada; . lunenfeld-tanenbaum research institute, Mount Sinai Health System, Toronto, Toronto, ON, Canada; . lunenfeld-tanenbaum research institute, Mount Sinai Health System, Toronto, Toronto, ON, Canada

## Abstract

**Background:**

Crohn's disease (CD) is a gastrointestinal disorder characterized by chronic inflammation. While increased oxidative stress is observed in established CD patients, it remains unknown whether a shift in redox status is present before the diagnosis of CD and whether it is correlated with changes in immune response and microbial composition. Our hypothesis is that oxidative stress plays a role in the development of CD, and it could be detected before the diagnosis of CD. Furthermore, it is likely to be correlated with systemic inflammation and alterations in gut microbiota composition.

**Aims:**

We aimed to assess the relationship between the serum redox status-related indicators, specifically amino acids ratios, with risk of CD onset and if this is further association with systemic inflammation marker, and gut microbiota composition.

**Methods:**

In the Genetic Environment Microbial Project (CCC-GEM), a cohort of first-degree relatives (FDRs) of CD patients was prospectively followed. Among them, we identified subjects who later developed CD, defined as pre-CD (n=69), and matched them at a 1:4 ratio with FDRs who remained disease-free (n=276). Serum levels at enrollment of cysteineglycine (reduced form) and cystineglycine (oxidized form) were quantified by mass spectrometry, and the cysteineglycine/cystineglycine ratio was used as an indicator of redox status. Conditional logistic regression assessed the association with CD, while partial Spearman regression evaluated its correlation with systemic inflammation, as indicated by c-reactive protein (CRP), and gut microbiota composition (determined by fecal 16S rRNA sequencing).

**Results:**

A decrease in the ratio indicates a shift in redox status toward oxidative stress. The cysteineglycine/cystineglycine ratio was negatively associated with the likelihood of developing CD (coefficient = -0.6188; *p* =0.0146), and it was also negatively correlated with CRP levels (coefficient = -0.177; *p* =0.00095). A list of taxa belonging to the phyla *Firmicutes* and *Actinobacteriota* were positively correlated with cysteineglycine/cystineglycine ratio (*p*≤0.05).

**Conclusions:**

This study is the first to report that when redox status shifts towards oxidative stress, as indicated by the cysteineglycine/cystineglycine ratio, the likelihood of CD increases. Furthermore, these markers also correlate with CRP levels and gut microbiota composition, indicating a loss of various taxa when the redox status shifts towards oxidative stress.

Submitted on behalf of the CCC-GEM consortium.

**Funding Agencies:**

CCC, CIHRHCT

